# The role of glial cells in mental illness: a systematic review on astroglia and microglia as potential players in schizophrenia and its cognitive and emotional aspects

**DOI:** 10.3389/fncel.2024.1358450

**Published:** 2024-02-14

**Authors:** Daniela Laricchiuta, Martina Papi, Davide Decandia, Anna Panuccio, Debora Cutuli, Maurizio Peciccia, Claudia Mazzeschi, Laura Petrosini

**Affiliations:** ^1^Department of Philosophy, Social Sciences and Education, University of Perugia, Perugia, Italy; ^2^Laboratory of Experimental and Behavioral Neurophysiology, IRCCS Santa Lucia Foundation, Rome, Italy; ^3^Department of Psychology, University Sapienza of Rome, Rome, Italy

**Keywords:** GFAP, Iba1, cognitive symptoms, inflammatory pathways, immune system

## Abstract

Schizophrenia is a complex and severe mental disorder that affects approximately 1% of the global population. It is characterized by a wide range of symptoms, including delusions, hallucinations, disorganized speech and behavior, and cognitive impairment. Recent research has suggested that the immune system dysregulation may play a significant role in the pathogenesis of schizophrenia, and glial cells, such as astroglia and microglia known to be involved in neuroinflammation and immune regulation, have emerged as potential players in this process. The aim of this systematic review is to summarize the glial hallmarks of schizophrenia, choosing as cellular candidate the astroglia and microglia, and focusing also on disease-associated psychological (cognitive and emotional) changes. We conducted a systematic review following the Preferred Reporting Items for Systematic Reviews and Meta-Analyses guidelines. We searched PubMed, Scopus, and Web of Science for articles that investigated the differences in astroglia and microglia in patients with schizophrenia, published in the last 5 years. The present systematic review indicates that changes in the density, morphology, and functioning of astroglia and microglia may be involved in the development of schizophrenia. The glial alterations may contribute to the pathogenesis of schizophrenia by dysregulating neurotransmission and immune responses, worsening cognitive capabilities. The complex interplay of astroglial and microglial activation, genetic/epigenetic variations, and cognitive assessments underscores the intricate relationship between biological mechanisms, symptomatology, and cognitive functioning in schizophrenia.

## 1 Introduction

Schizophrenia is a complex, multi-dimensional, and enduring psychiatric disorder characterized by a broad spectrum of clinical manifestations that include positive (hallucinations, delusions, disorganized speech, and thought disturbances), negative (reduction or absence of typical emotional, motivational, or interest-related behaviors), and cognitive (impairment in working memory, verbal memory, attention, and executive functions) symptoms (Owen et al., 2016; Correll and Schooler, 2020). Typically emerging during late adolescence to early adulthood, schizophrenia exhibits gender-specific onset patterns, with males often experiencing an earlier onset (late adolescence to early twenties) compared to females (early twenties to early thirties) (Hollis et al., 2008; McGrath et al., 2008). Despite subtle changes in cognition and social relationships preceding formal diagnosis by several years, the diagnosis is often delayed, and patients already display significant brain changes in terms of structure, neurochemistry, and connectivity (Millan et al., 2016; Rodrigues-Neves et al., 2022). The clinical presentation, trajectory, and prognosis of schizophrenia are highly heterogeneous, marked by recurrent and challenging-to-predict relapses, often leading to a chronic course of illness (Owen et al., 2016). The heterogeneity of symptoms and their late recognition make outlining a pathophysiological profile challenging. Despite extensive research, specific structural and functional dysregulations, prodromal and unique to schizophrenia, have not been conclusively identified. Patients with schizophrenia exhibit reductions in the volume of the caudate nucleus, thalamus, and prefrontal cortex (PFC) (Lewis, 2012; Haijma et al., 2013; Meyer-Lindenberg and Tost, 2014). Dysregulation of dopaminergic neurotransmission is a key feature, with dopamine excess in subcortical limbic area linked to positive symptoms (Howes and Kapur, 2009; Walter et al., 2009), and dopamine deficiency in the PFC associated with negative and cognitive symptoms (Fusar-Poli et al., 2011). Cognitive deficits also correlate with impaired glutamatergic neurotransmission (Kantrowitz and Javitt, 2010; Barch and Ceaser, 2012).

Interestingly, a comprehensive genome-wide association study in schizophrenia has emphasized common variations in genes encoding for the glutamate receptors, voltage-dependent calcium channel proteins, and dopamine receptors D2 (DRD2), supporting the role of glutamatergic and dopaminergic signaling disruption in schizophrenia pathogenesis (Schizophrenia Working Group of the Psychiatric Genomics Consortium, 2014). Moreover, the same working group found associations between schizophrenia and genes with important roles in immunity, in line with observational and clinical studies highlighting the connections between immune and inflammatory processes in psychiatric disorders (Benros et al., 2012; Smyth and Lawrie, 2013; Kroken et al., 2018; Hughes and Ashwood, 2020). More recently, research on psychiatric and neurological disorders has increasingly pointed to the neural-immune interface as potential source of disease markers, thereby enabling the identification of new neurobiological targets (Afridi et al., 2021; Chen et al., 2023).

The neuroimmune system is implied in a wide variety of brain functions, such as regulation of the neuroinflammatory status, modulation of neurotransmission and neuroplasticity, central nervous system (CNS) development, and response to injuries (Dantzer, 2018; O’Reilly and Tom, 2020). The neuroimmune system comprises glial cells like astrocytes, microglia, oligodendrocytes, and NG2 positive cells (Takahashi and Sakurai, 2013). Specifically, astrocytes encompass diverse cell populations, primarily categorized based on morphological characteristics and/or marker expressions (Zhang and Barres, 2010). Astrocytes provide metabolic support to neurons, regulate extracellular water and electrolyte balance, and synthesize an extensive array of extracellular matrix proteins and growth factors (De Keyser et al., 2008). Moreover, astrocytes have a pivotal role in synaptic formation, efficacy, and plasticity (Barker and Ullian, 2010; Kucukdereli et al., 2011; Suzuki et al., 2011), and release neurotransmitters, including D-serine, ATP, and glutamate (Hamilton and Attwell, 2010), exerting a direct influence on brain homeostasis. In brain pathologies, activated astrocytes have the capacity to modulate the immune responses producing proinflammatory cytokines [such as interleukin (IL)-6, IL-1β, tumor necrosis factor (TNF)-α, and interferon γ], reactive oxygen species (ROS), and matrix metalloproteases, contributing thus to the neuroinflammatory response (De Keyser et al., 2008).

Another essential type of neuroimmune heterogeneous cells is the microglia, categorized in subpopulations according to the expression of particular gene clusters. It presents distinct transcriptomic profiles with brain region specificity that change over time to functionally respond to particular demands of different developmental phases (Matcovitch-Natan et al., 2016; Thion et al., 2018). Acting as phagocytes, microglial cells also produce proinflammatory cytokines along with ROS (Woodburn et al., 2021). Microglia plays a key role in the synaptic pruning, contributes to the proper establishment and maturation of neural circuits, and influences growth and survival of dopaminergic neurons in the context of inflammation (Saijo and Glass, 2011; Coomey et al., 2020).

The regulation of astrocyte and microglia activity is crucial for physiological brain functioning and homeostasis, and any disruption in these processes may have detrimental impact on mental health. Astrocytes and microglia are able to eliminate or strengthen cells and synapses, functions of high relevance in the onset of schizophrenia and its clinical progression. This systematic review focused on recent findings (from the past 5 years) exploring the involvement of astrocytes and microglia evaluated by using neurobiological, morphological, and/or genetic and epigenetic analyses in patients with schizophrenia, aiming to better clarify (i) astroglial and microglial cellular targets in the disease, and (ii) their relationship with psychological (cognitive and emotional) correlates.

## 2 Materials and methods

### 2.1 Protocol

**FIGURE 1:**
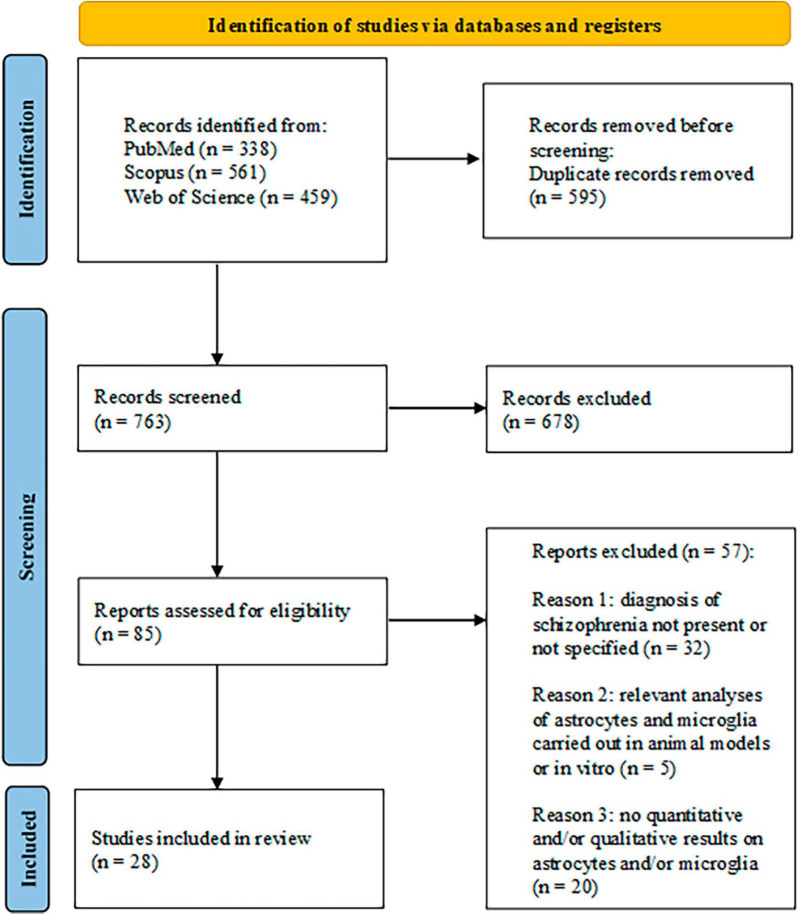
Search flow diagram based on PRIMA guidelines.

This systematic review was created using the Preferred Reporting Items for Systematic Reviews and Meta-Analyses (PRISMA) guidelines (Page et al., 2021). In Figure 1, the PRISMA flowchart of the study selection was reported.

### 2.2 Search strategy and study selection

A systematic literature search was performed on three databases: PubMed, Scopus, and Web of Science to identify articles published in the 5 years prior to the research date (28 April 2023), focused on the following areas of interest: “schizophrenia” and “astrocytes and microglia alterations.” After keywords selection, the search was performed within “Title and abstract” in PubMed and Scopus, and within “Topic” in Web of Science. In the following, advanced searches for each database are reported:

– PubMed advanced search: (((”schizophrenia”) AND ((”microglia” OR “Iba1” OR “microglial cell*”) OR (”astroglial cell*” OR “astroglia” OR “GFAP” OR “astrocytes”))) NOT “review”).
– Scopus advanced search: (((”schizophrenia”) AND ((”microglia” OR “Iba1” OR “microglial cell*”) OR (”astroglial cell*” OR “astroglia” OR “GFAP” OR “astrocytes”))) AND NOT “review”).
– Web of Science advanced search: (((”schizophrenia”) AND ((”microglia” OR “Iba1” OR “microglial cell*”) OR (”astroglial cell*” OR “astroglia” OR “GFAP” OR “astrocytes”))) NOT “review”).

The screening was independently performed by three different authors.

### 2.3 Inclusion and exclusion criteria

The PICOS model was used to determine the inclusion criteria:

– P (population): “individuals diagnosed with schizophrenia”;
– I (intervention): not applicable;
– C (comparators): “individuals without a psychiatric diagnosis (i.e., control and placebo groups)”;
– O (outcome): “astrocytes and microglia evaluations”;
– S (study design): “observational studies, cohort studies, clinical trials, cross-sectional studies, and case-control studies.”

All human studies were included despite the study design. On the contrary, all studies including animal models or *in vitro* and *in silico* studies were excluded. In order to properly adhere to the research question, we excluded articles that did not involve patients with schizophrenia whose diagnosis was reported and involved a validated method. Additionally, we excluded articles that analyzed astrocyte and microglia alterations in patients with schizophrenia, but not considering at least one of the following methodologies:

– Neurobiological analyses;
– Morphological analyses;
– Genetic analyses;
– Epigenetic analyses.

### 2.4 Data extraction

After defining inclusion and exclusion criteria and having completed the selection of studies, data were extracted and summarized in the Tables, reporting the following information:

– Description of all selected studies including *in vivo*/*ex vivo*, sample details, diagnosis, medical treatments, and exclusion criteria (Table 1);
– Methodologies (Table 2) and results (Table 3) of investigations on astrocytes including neurobiological and morphological analyses, genetic and epigenetic analyses, and psychological assessment;
– Methodologies (Table 4) and results (Table 5) of investigations on microglia including neurobiological and morphological analyses, genetic and epigenetic analyses, and psychological assessment;
– Methodologies (Table 6) and results (Table 7) of investigations on both astrocytes and microglia including neurobiological and morphological analyses, genetic and epigenetic analyses, and psychological assessment.

**TABLE 1:** Summary of the sample description of all studies.

References	*In vivo*/ *ex vivo*	Specific description of sample	Diagnosis	Medical treatment	Exclusion criteria
Hagenauer et al., 2018	*Ex vivo*	5 post-mortem human cortical tissue datasets containing: Microarray data from 4 datasets:−22 schizophrenia patients and 71 controls (age: 52 ± 15; 27% F)−14 schizophrenia patients and 18 controls (age: 46 ± 15; 45% F) −23 schizophrenia patients and 19 controls (age: 70 ± 19; 45% F) −27 schizophrenia patients and 27 controls (age: 45 ± 17; 17% F)RNA-Seq data from one dataset: −263 schizophrenia patients and 285 controls (age: 65 ± 18; 41% F).	DSM-IV-TR.	–	–
O’Donovan et al., 2018	*Ex vivo*	16 schizophrenia subjects (age: 45 ± 12, ranged 20–67; 14–M; 2–F). A total of 16 controls (age: 43 ± 10, ranged 23–60; 14–M, 2–F).	For all post-mortem human cases DSM-IV diagnoses based on the review of available medical records, autopsy reports, and interviews with family using the Structured Clinical Interview for DSM-IV (SCID).	Medication on: 13 schizophrenia patients Medication off: 1 schizophrenia patient Unknown: 2 schizophrenia patients.	Data analysis: all data sets were tested for normal distribution (D’Agostino and Pearson omnibus normality test) and homogeneity of variance (*F*-test). Outliers >2 standard deviations from the mean were excluded.
López-González et al., 2019	*Ex vivo*	14 controls (all M; age at death: 71 ± 8 years) 14 chronic schizophrenia patients (long term illness; all M; age at death: 76 ± 11 years).	DSM-IV and ICD-10. Study included the following schizophrenia diagnoses: chronic residual schizophrenia (11), chronic paranoid schizophrenia (2), and chronic disorganized schizophrenia (1).	3 patients were being medicated with first-generation antipsychotics (21.4%), 7 patients were being medicated with second- generation antipsychotics (50%), and 4 schizophrenia patients were antipsychotic-free (28,6%).	Alzheimer’s disease-related pathology stages IV-VI of Braak and Braak were not included in the present study.
Adorjan et al., 2020	*Ex vivo*	8 schizophrenia patients (3–M, 5–F) 6 controls (2–M, 4–F).	DSM-III and DSM-IV.	5 schizophrenia patients received anti-psychotic treatment. A total of 2 schizophrenia patients received no or minimal medication.	–
Amoah et al., 2020	*Ex vivo*	Brain total RNA samples from the orbitofrontal cortex of schizophrenia patients (29; 20–M and 9–F) and controls (25; 21–M and 4–F).	DSM-IV.	Antipsychotic treatment: fluphenazine.	Samples with an RNA integrity number lower than 6.5 were excluded.
Chan et al., 2020	*Ex vivo*	Methylome-wide sequencing data from 759 schizophrenia patients and 738 controls (Swedish). In addition, 3 methylation datasets: Montano et al.: DNA from blood of 689 schizophrenia patients and 645 controls; Hannon-1: DNA from blood of 353 schizophrenia patients and 322 controls; Hannon-2: methylation profiles from blood of 414 schizophrenia patients and 433 controls.	Montano: DSM-IV. Hannon-1: ICD-10. Hannon-2: ICD-10, and DSM-IV.	Antipsychotic medication	–
Hill et al., 2021	*Ex vivo*	35 schizophrenia patients (age: 42.6 ± 7.6; 26–M, 9–F). A total of 35 controls (age: 44.2 ± 7.6; 26–M, 9–F).	DSM-IV.	Antipsychotic dose. Prescribed mood stabilizers: 11 schizophrenia patients. Prescribed antidepressant: 9 schizophrenia patients.	–
Karpiński et al., 2020	*Ex vivo*	7 datasets. A total of 273 schizophrenia patients and 302 controls.	DSM-III (patients with residual schizophrenia), DSM-IV, Diagnostic Instrument for Brain Studies, interviews with family members.	–	Samples with the AffyRNADegradation estimates dk ≤0.45 were excluded from subsequent analyses.
Kindler et al., 2020	*Ex vivo* and *in vivo*	Dorsolateral prefrontal cortex (DLPFC): 37 schizophrenia patients and 37 controls. Middle gyrus: 34 schizophrenia patients and 35 controls. Living cohort: 96 schizophrenia or schizoaffective patients and 81 controls.	DSM-IV.	All schizophrenia patients were receiving antipsychotic medication (95% receiving second-generation antipsychotics) for at least 1 year prior to participation. Mean daily dose of antipsychotic medication for each patient was converted to approximate daily mean CPZ milligram equivalents.	concurrent DSM-IV Axis I diagnosis other than schizophrenia or schizoaffective disorder for patients or any personal history or first-degree relative with a DSM-IV Axis I disorder for controls. For all participants: history of uncontrolled diabetes or cardiovascular disease including hypertension, central nervous system infection, recent alcohol/substance abuse (within the past 5 years), head injury with loss of consciousness, epileptic seizures, structural brain abnormalities, developmental disorders, and/or mental retardation.
Petrasch-Parwez et al., 2020	*Ex vivo*	Brain tissue from 34 age-matched individuals. A total of 17 schizophrenia patients (11–M, 6–F). A total of 17 controls (13–M, 4–F).	DSM-IV.	-	No known neurological or psychiatric disorder in controls individuals.
Rodrigues-Amorim et al., 2020	*In vivo*	42 schizophrenia patients (age: 41.10 ± 14.41; 26–M and 16–F). A total of 40 controls (age: 44.96 ± 14.95; 25–M and 15–F).	DSM-V.	Schizophrenia patients: 6 were drug näive, 33 were treated with Cilostazol (CSZ), 8 with aripiprazole, 8 with risperidone, 8 with olanzapine, 9 with clozapine. Each schizophrenia patient had been treated with only one antipsychotic (risperidone, aripiprazole or olanzapine), except clozapine-treated patients, who had not responded before to two different types of antipsychotics.	Individuals with other psychiatric or neurological disorders, traumatic brain injury, diabetes or history of substance abuse, pregnant or breastfeeding women.
Uranova et al., 2020	*Ex vivo*	21 schizophrenia patients (age: 56.3 ± 16.8; 11–M, 10–F). A total of 12 schizophrenia patients in the subgroup with predominantly positive symptoms (SPSS), 9 patients in the subgroup with predominantly negative symptoms (SPNS). A total of 20 controls (age: 58.3 ± 12.6; 12–M, 8–F).	DSM-IV TR.	Antipsychotic medication (CPZ equivalents were estimated for the patient’s last 30 days).	Alcohol, drug abuse, other neuropsychiatric disorders. Cases were excluded from the study by an experienced neuropathologist if there was evidence for neurological damage, neoplastic, vascular and neurodegenerative changes.
Zhang et al., 2020	*Ex vivo*	35 schizophrenia patients. Schizophrenia patients who committed suicide: 7 (age: 39, range: 24–45; 4–M, 3–F). Schizophrenia patients died of other causes: 28 (age: 44, range: 19–59; 22–M, 6–F). A total of 34 controls (age: 45, range: 31–60; 25–M, 9–F).	DSM-IV.	Antipsychotics, recorded as fluphenazine equivalents in a lifetime dosage.	Significant structural brain pathology on post-mortem examination or by ante- mortem imaging, history of significant focal neurological signs ante- mortem, history of a central nervous system disease that could be expected to permanently alter gene expression, documented IQ < 70, poor RNA quality as indicated by an RNA integrity value of lower than 7. Additional exclusion criteria for controls, including age under 30 and substance abuse within 1 year before death or significant alcohol-related changes in the liver.
Corley et al., 2021	*In vivo*	1238 Irish schizophrenia patients and controls (age: 18–72, mean 41.15, s.d. 12.84; 740–M, 489–F) composed of 908 patients with either a diagnosis schizophrenia and schizoaffective disorder (*n* = 676) and 330 controls (age: 18–65).	Structural Clinical Interview for the DSM-IV (SCID).	Antipsychotic.	No history of major mental health issues, intellectual disability or acquired brain injury in control individuals.
Jeon et al., 2021	*In vivo*	21 first-episode schizophrenia patients. A total of 16 M, 5 F (age: 22.33, s.d. A total of 5.29; 16–M, 5–F; ethnicity: 2 black, 18 white, 1 other). A total of 10 controls. A total of 5 M, 5 F (age: 21.60, s.d. 3.37; 5–M, 5–F, ethnicity: 0 black, 5 white, 5 other).	DSM-V.	Antipsychotic exposure at baseline mean 2.95, s.d. 3.11. Patients were required to have <14 days of lifetime exposure to antipsychotics.	Any participant whose 6-month diagnoses were bipolar or major depressive disorder with psychoses, as well as suspected drug-induced psychoses. Control individuals were screened to ensure to have no personal history of mental illness and no family history of psychotic disorder. None of the participants had a significant head injury, major medical illness, or MRI contraindications.
Ranganathan et al., 2021	*In vivo*	24 patients with early psychosis (Primary psychotic disorder and 5 years onset of psychosis) and 12 controls (Age: 18–65; 20 M–4 F).	Structured diagnostic interview for DSM IV Axis I disorders (SCID I)	All except two patients were on oral or depot antipsychotic medications at the time of sample collection with a mean (SD) dose of 10.73 (9.66) mg in olanzapine equivalents.	Other current major psychiatric diagnoses that require pharmacological treatment. Current substance dependence. Serious medical or neurological illness or treatment for a medical disorder that could interfere with study. History of significant head injury/trauma. Women who are pregnant, nursing. History of treatment resistance or current treatment with clozapine.
Shimamoto-Mitsuyama et al., 2021	*Ex vivo*	Post-mortem brain tissues used in lipid analysis: 15 schizophrenia patients (age: 58 ± 14; 8–M, 7–F) and controls (age: 57 ± 13; 8–M, 7–F). Gene expression analysis: 95 schizophrenia patients and 91 controls.	DSM-IV and the Diagnostic Instrument for Brain Studies.	Antipsychotic, CPZ equivalents.	Any history of psychiatric illness.
Uranova et al., 2021	*Ex vivo*	Brain tissue from 21 patients with chronic schizophrenia (26.7 ± 10.8 years, hospitalized) and 20 controls.	ICD-10 and DSM-IV.	Patient treated with typical and atypical neuroleptics (chlorpromazine (CPZ) equivalents), except for 3 patients.	Cancer and cases of comorbid alcoholism or drug abuse.
Di Biase et al., 2022	*In vivo*	1849 participants from three datasets. Two datasets combined in single cohort (1267 comprising 140 schizophrenia patients age: 22.78 ± 3.83; 48–F) and 1127 controls (age: 28.50 ± 3.83; 598–F). Third dataset 520 comprising 335 schizophrenia patients (age: 39.70 ± 10.81; 100–F) and 185 controls (age: 41.05 ± 14.02; 94–F).	DSM-V.	Chlorpromazine-equivalent antipsychotic medication.	Exclusion criteria for each respective dataset are described in the relative studies: HCP-Young adult (Van Essen et al., 2013); HCP-Psychosis (Lewandowski et al., 2020) and ASRB (Loughland et al., 2010).
Endres et al., 2022	*In vivo*	70 schizophrenia patients (37 F, 33 M; age: 32.7 ± 12.9; 33–M, 37–F).	ICD-10.	Overall psychopharmacological treatment (including all participants of the study): 113 patients of which 56 antidepressant, 18 typical antipsychotic, 82 antipsychotics overall, 15 lithium, 23 anticonvulsants, 34 benzodiazepines.	Patients with established neurologic disorders (e.g., acute infectious encephalitis)
Huang et al., 2022	*In vivo*	78 schizophrenia patients (age: 24.73 ± 5.10; 45–M, 33–F). A total of 71 controls (age: 26.30 ± 5.10; 33–M, 38–F).	ICD-10.	–	Previous history of mental retardation, encephalitis, epilepsy and other organic brain diseases or other neurological diseases. Patients with diseases related to immune and endocrine systems or other systemic diseases. Abuse of drugs or psychoactive substances. Patients with a history of cranio-cerebral trauma. Impulsive or uncooperative patients.
Jiang et al., 2021	*In vivo*	Combined dataset: 469 schizophrenia patients, 481 controls. Independent cohort: 182 schizophrenia patients, 351 controls.	Structured Clinical Interview for Diagnosis for DSM-IV or DSM-IV TR (SCID).	Antipsychotic.	–
Pinjari et al., 2022	*In vivo*	106 schizophrenia patients (age: 32.90 ± 12.28; 76–M, 30–F; ethnicity: 55 African American, 33 Caucasian, 16 Hispanic, 2 Asian).	DSM-V. The Mini International Neuropsychiatric Interview version 5 (MINI) used to confirm the diagnosis of schizophrenia.	Antipsychotic treatment. Mean chlorpromazine equivalent: 413.94 ± 287.07.	Cognitive disorders including and not limited to pervasive developmental disorder, dementia, delirium, current suicidal and homicidal ideations, urine drug screen positive for psycho-stimulants such as cocaine, amphetamines and ecstasy, primary inflammatory conditions including any infection, neoplasm, autoimmune diseases, non-steroidal anti-inflammatory drug use, current or anticipated corticosteroid use, recent use of warfarin or any anticoagulant.
Schmitt et al., 2022	*Ex vivo*	11 schizophrenia patients (50.9 ± 3.7 years). A total of 11 age-matched controls (54.5 ± 7.1 years). All males. Ethnic background of both groups was white European.	DSM-IV and ICD-10.	All patients were treated with antipsychotic during most illness.	Neurological diseases requiring treatment or that may have influenced cognitive tests (e.g., stroke with aphasia); history of recurrent seizures lifetime severe craniocerebral trauma with loss of consciousness; history of alcohol or substance abuse disorders; and type 2 diabetes with a free plasma glucose level greater than 200 mg/dL.
Vikhreva and Uranova, 2022	*Ex vivo*	10 attack-like progressive schizophrenia patients (age 53 ± 12.2 years; 5–M, 5–F). A total of 9 paranoid continuous schizophrenia patients (age 58.8 ± 17.3 years; 6–M, 3–F). A total of 20 controls.	ICD-10.	The possible influence of neuroleptic therapy on the study parameters was assessed in terms of the CPZ equivalent.	No mental or neurological pathology in control individuals.
Jenkins et al., 2023	*Ex vivo*	Brain tissue from 62 schizophrenia patients and 62 controls (47 M–15 F). Diagnostic group: 47.7 ± 12.7 years old, 46 white and 16 black.	Review of all available medical records in tandem with structured interviews with individual family members. DSM-IV.	Medications at time of death in diagnostic group: 54 patients were taking antipsychotic, 27 antidepressant, 24 benzodiazepine/anticonvulsant.	–
Yu et al., 2023	*Ex vivo*	Transcriptional expression data concerning the post-mortem DLPFC (Brodmann areas 9 and 46):schizophrenia patients (160–M, 93–F) and controls (154–M, 113–F).	DSM-IV.	–	–
Zong et al., 2023	*In vivo*	42 drug-naive first-episode schizophrenia patients (age: 24.86; 27–M, 15–F). A total of 38 age-, gender- and education-matched controls (age: 24.76; 25–M, 13–F).	Structured Clinical Interview for DSM-IV-TR (SCID). SCID-non-patient edition to scan controls to confirm that they had no history of neurological and psychiatric disorders.	Drug-naïve patients. Antipsychotic therapy: starting daily dose of risperidone was 1 or 2 mg and then a slow titration protocol after 1 week. Specifically, increased risperidone dose at 1-week intervals until the patients had clinical improvement. For those patients who did not exhibit improvement after 4 weeks, they reach a maximum dose of 6 mg/day. All 42 patients were given risperidone monotherapy for 8 weeks. Mood stabilizers or antidepressants were not allowed to use.	–

**TABLE 2:** Summary of the methodological approach of studies performing analyses on astrocytes.

Methods	Astrocytes		Psychological assessment
References	Neurobiological and morphological analyses	Genetic and epigenetic analyses	
O’Donovan et al., 2018	Laser capture microdissection: analyses of enriched population of astrocytes and pyramidal cells, layer II-III-V-VI of the DLPFC.	Quantitative PCR of: ENTPDs, 5′nucleotidase, ENT1, ADA, ADK, A1R, and A2AR.	No psychological assessment was used.
Amoah et al., 2020	No neurobiological analyses were performed.	Real-Time Quantitative Reverse Transcription PCR (qRT-PCR) in the orbitofrontal cortex.	No psychological assessment was used.
Chan et al., 2020	No neurobiological analyses were performed.	Methylome-wide association study conducted for schizophrenia patients’ blood through existing sequencing-based methylation data; comparison between MWAS and existing array-based schizophrenia patients methylation studies in blood.	No psychological assessment was used.
Rodrigues-Amorim et al., 2020	Blood samples collection; quantitative immunoblotting of β-III tubulin, Nf-L and GFAP; sandwich ELISA kit for GFAP.	No genetic or epigenetic analyses were performed.	Cognitive evaluation: PANSS.
Jeon et al., 2021	7 T magnetic resonance spectroscopy at the anterior cingulate cortex to quantify myo-inositol levels at baseline and after treatment.	No genetic or epigenetic analyses were performed.	Cognitive evaluation: PANSS, SOFAS. Emotional evaluation: CDSS.
Ranganathan et al., 2021	Exosomal analysis through exosome isolation kit; Nanoparticle tracking analysis; Western blot analysis of: CD9, GFAP, synaptophysin, and a-II-Spectrin.	No genetic or epigenetic analyses were performed.	Cognitive evaluation: PANSS.
Endres et al., 2022	(18F)-fluorodeoxyglucose positron emission tomography; Immunoblot for the analysis of autoantibodies against intracellular paraneoplastic autoantibodies; Cerebrospinal fluid and serum samples was collected to analyze anti-neural autoantibodies against cell surface antigens through fixed cell-based assay; Indirect immunofluorescence on unfixed murine brain was used for tissue-based assay.	No genetic or epigenetic analyses were performed.	No psychological assessment was performed.
Huang et al., 2022	Blood samples collection; ELISA kit for GFAP, NGF, BDNF, IL-6, TNF-α, S100β.	No genetic or epigenetic analyses were performed.	Cognitive evaluation: PANSS, MCCB (SoP, VeL, RPS, ViL).
Pinjari et al., 2022	Plasma collection; ELISA kit for P-selectin, E-selectin, L-selectin; Human S100B ELISA kit for S100B; standard 24-panel Bio-Plex ProTM human inflammation assay for IL-6.	No genetic or epigenetic analyses were performed.	No psychological assessment was performed.
Schmitt et al., 2022	Stereological analyses of whole hippocampus with a focus on the CA4 and the dentate gyrus; analyses of neurons, oligodendrocytes, astrocytes and granule cells.	No genetic or epigenetic analyses were performed.	No psychological assessment was used.

**TABLE 3:** Summary of the results of studies performing analyses on astrocytes.

Results	Astrocytes		Psychological assessment
References	Neurobiological and morphological analyses	Genetic and epigenetic analyses	
O’Donovan et al., 2018	No neurobiological results were detected	Significant decrease in enriched population of astrocytes of ENTPD1, ENTPD2 mRNA levels in schizophrenia patients.	No psychological evaluation was performed.
Amoah et al., 2020	No neurobiological results were detected	Increase of miR-223 in the orbitofrontal cortex of schizophrenia patients and bipolar disorder patients with psychosis; a positive association was found between SERPINA3 and miR-223 level.	No psychological evaluation was performed.
Chan et al., 2020	No neurobiological results were detected	22 thoroughly significant loci; two most-significant site located in: MFN2, ALDH1A2.	No psychological evaluation was performed.
Rodrigues-Amorim et al., 2020	Increase of GFAP levels in schizophrenia patients compared to healthy controls; in clozapine-treated schizophrenia patients, the levels of GFAP were moderately higher compared to other groups.	No genetic or epigenetic results were detected.	The positive and the negative subscales of PANSS did not show statistically significant outcome within groups; considering the treatments, it was seen a difference between schizophrenia subgroups on risperidone, olanzapine, aripiprazole.
Jeon et al., 2021	Lower levels of myo-inositol at baseline in schizophrenia patients compared to healthy controls; after 6 months of antipsychotic treatment the difference was not detected anymore.	No genetic or epigenetic results were detected.	Negative correlation between myo-inositol concentration and PANSS-8 scores, a less marked association with SOFAS, no association with CDSS.
Ranganathan et al., 2021	All schizophrenia patients showed positive immunostaining with polyclonal anti-GFAP antibody, contrary to the control group; increased in CD9 normalized concentration of GFAP and decreased in CD9 normalized a-II- Spectrin concentration in schizophrenia patients.	No genetic or epigenetic results were detected.	PANSS total and subscale scores did not have a significant association with GFAP concentration in schizophrenia patients.
Endres et al., 2022	In one patient was detected low titer anti-GFAP autoantibodies in serum, not in cerebrospinal fluid.	No genetic or epigenetic results were detected.	No psychological evaluation was performed.
Huang et al., 2022	No significant difference in the level of GFAP between the patient group and healthy controls; increase in TNF-α, IL-6, S100β and NGF in the schizophrenia patients compared to controls; decrease of BDNF in the schizophrenia patients compared to controls.	No genetic or epigenetic results were detected.	SoP, VeL, RPS and ViL T-scores in schizophrenia patients were lower than the healthy control group; negative correlation between T fraction of ViL and GFAP, TNF-α, S100β, NGF, BDNF.
Pinjari et al., 2022	Positive correlation between soluble P-selectin with S100B and IL-6.	No genetic or epigenetic results were detected.	No psychological evaluation was performed.
Schmitt et al., 2022	No significant differences in the number and the density of astrocytes, neurons and granular neurons in CA4 of schizophrenia patients; decrease in number and density of oligodendrocytes in CA4 of schizophrenia patients.	No genetic or epigenetic results were detected.	No psychological evaluation was performed.

**TABLE 4:** Summary of the methodological approach of studies performing analyses on microglia.

Methods	Microglia		Psychological assessment
References	Neurobiological and morphological analyses	Genetic and epigenetic analyses	
Adorjan et al., 2020	Immunohistochemistry with primary antibodies: anti- calretinin, anti-neuropeptide Y, anti-ionized calcium-binding adapter molecule 1 (Iba1), anti-transmembrane protein 119 (TMEM119) in caudate nucleus.	Reverse-Transcriptase qPCR for: GAPDH, PPIA, ACTB, SDHA, SNCA, TBP, UBC in superior frontal gyrus.	No psychological assessment was used.
Hill et al., 2021	Immunoblotting: protein expression of CX3CR1 and ADAM10 were quantified in DLPFC; ELISA kit for SNAP-25.	Droplet Digital PCR for CX3CR1, ADAM10.	No psychological assessment was used.
Petrasch-Parwez et al., 2020	Immunohistochemistry for Iba1 in the anterior midcingulate cortex; quantification of microglia cells with cresyl violet-staining.	No genetic or epigenetic analyses were performed.	No psychological assessment was used.
Uranova et al., 2020	Transmission electron microscopy; morphometry of microglia and adjacent oligodendrocytes in BA10.	No genetic or epigenetic analyses were performed.	Cognitive evaluation: SANS, SAPS to divide subjects in SPPS and SPNS.
Jiang et al., 2021	Structural MRI.	DNA extraction from blood or saliva; extraction of 12q24 quantitative trait locus, individuation of overlapping single nucleotide polymorphism; pICA.	No psychological assessment was used.
Vikhreva and Uranova, 2022	Morphometry of microglia in the PFC through electron microscopy.	No genetic or epigenetic analyses were performed.	No psychological assessment was used.
Jenkins et al., 2023	No neurobiological analyses were performed.	Quantitative PCR in PFC: analyzed mRNA levels for C4, C1q, C3, CR3, Axl, MerTK, CD68, TREM2, DAP12, THIK-1, P2Y12, and TSPO.	No psychological assessment was used.
Yu et al., 2023	No neurobiological analyses were performed.	Transcriptional expression data concerning DLPFC; different expression gene analysis; RNA-seq analysis.	No psychological assessment was used.

**TABLE 5:** Summary of the results of studies performing analyses on microglia.

Results	Microglia		Psychological assessment
References	Neurobiological and morphological analyses	Genetic and epigenetic analyses	
Adorjan et al., 2020	No recognizable histological patterns in schizophrenia patients; the evaluation of Iba1 and TMEM119-immunopositive cells did not showed between schizophrenia patients and healthy controls.	No statically significant differences were found in the transcript levels of Iba1 and TMEM119 between schizophrenia patients and healthy controls.	No psychological evaluation was performed.
Hill et al., 2021	Significant decrease in mean fractalkine levels in schizophrenia patients, unchanged in bipolar disorder; no difference was found in CX3CR1 between groups.	No significant difference was found in CX3CL1 and CX3CR1 mRNA expression between schizophrenia and bipolar disorder group; it was found a significant correlation between protein level of CX3CR1 and SNAP-25 in control group, not reaching statical significance in schizophrenia patients.	No psychological evaluation was performed.
Petrasch-Parwez et al., 2020	No statistically significant difference in microglia density between schizophrenia patients and healthy controls, the mean density was lowest in schizophrenia patients but it did not reach significance; microglial density was significantly lateralized toward the right anterior midcingulate cortex in schizophrenia patients and in bipolar disorder group compared to controls.	No genetic or epigenetic results were detected.	No psychological evaluation was performed.
Uranova et al., 2020	Increase of Vv of lipofuscin granules, Vv and the number of vacuoles of endoplasmic reticulum in microglia in SPPS; in SPPS the area of nucleus was negatively correlated with age and age at disease onset; it was found a correlation in SPPS between Vv, the number of mitochondria in microglia and the number of vacuoles in microglia, as well as with Vv and number of mitochondria in oligodendrocytes; correlation in SPPS between Vv of mitochondria in microglia and Vv, number of vacuoles in oligodendrocytes.	No genetic or epigenetic results were detected.	No psychological evaluation was performed.
Jiang et al., 2021	Negative correlation between the mPFC gray matter—insula component and genes in 12q24 QTL.	Highest contributing single nucleotide polymorphisms in validation cohort: WSCD2, SART3, CMKLR1, ISCU, and TMEM119.	No psychological evaluation was performed.
Vikhreva and Uranova, 2022	Microglial ultrastructure was heterogenous both in schizophrenia patients and in healthy controls, more transitional forms were observed in the schizophrenia patients. Increase of microglial density, in area of vacuoles in attack-like progressive schizophrenia, microglial soma, nuclear areas and the number of mitochondria correlated negatively with age and disease duration; decrease in Vv, number of mitochondria and an increase in Vv, the number, the area of lipofuscin granules in both clinical groups.	No genetic or epigenetic results were detected.	No psychological evaluation was performed.
Jenkins et al., 2023	No neurobiological results were detected.	Increased in mean C4, C1q, Axl, MerTK, CD68 mRNA levels in the schizophrenia patients; there were no statical differences in TREM2 and DAP12 between schizophrenia patients and healthy controls; increased in relative levels of THIK-1 mRNA and decrease in mRNA levels for P2Y12 in schizophrenia patients.	No psychological evaluation was performed.
Yu et al., 2023	No neurobiological results were detected.	Up-regulation of 120 genes, down-regulation of 35 genes in the female groups, specifically enriched in schizophrenia female patients; these genes were related to midbrain dopaminergic and GABA-ergic neurons and PFC C3 microglia.	No psychological evaluation was performed.

**TABLE 6:** Summary of the methodological approach of studies performing analyses on both astrocytes and microglia.

Methods	Astrocytes		Microglia		Psychological assessment
References	Neurobiological and morphological analyses	Genetic and epigenetic analyses	Neurobiological and morphological analyses	Genetic and epigenetic analyses	
Hagenauer et al., 2018	No neurobiological analyses were performed.	Database of transcripts, enriched in: astrocytes, endothelial cells, mural cells, microglia, immature and mature oligodendrocytes, interneurons, projection neurons; BrainInABlender; profiling of cell type specific gene expression in psychiatric human microarray datasets.	No neurobiological analyses were performed.	Database of transcripts, enriched in: astrocytes, endothelial cells, mural cells, microglia, immature and mature oligodendrocytes, interneurons, projection neurons; BrainInABlender; profiling of cell type specific gene expression in psychiatric human microarray datasets.	No psychological assessment was used.
López-González et al., 2019	No neurobiological analyses were performed.	RNA extracted from DLPFC; reverse transcription quantitative PCR for GUSB, HPRTT1, C1QL1, C1QTNF7, C3AE1, CSF1R, CSF3R, TLR4, TLR7, IL8, IL1B, IL6, IL6ST, TNFα, TNFRSF1A, IL10, IL10RA, IL10RB, TGB1, TGB2, AIF1, CD68, GFAP.	No neurobiological analyses were performed.	RNA extracted from DLPFC; reverse transcription quantitative PCR for GUSB, HPRTT1, C1QL1, C1QTNF7, C3AE1, CSF1R, CSF3R, TLR4, TLR7, IL8, IL1B, IL6, IL6ST, TNFα, TNFRSF1A, IL10, IL10RA, IL10RB, TGB1, TGB2, AIF1, CD68, GFAP.	Cognitive evaluation: PANSS.
Karpiński et al., 2020	No neurobiological analyses were performed.	Data analysis based on gene expression datasets from seven brain regions: associative striatum, hippocampus, parietal cortex, superior temporal cortex, nucleus accumbens, anterior cingulate cortex, DLPFC; Gene Set Enrichment Analyses; BrainInABlender.	No neurobiological analyses were performed.	Data analysis based on gene expression datasets from seven brain regions: associative striatum, hippocampus, parietal cortex, superior temporal cortex, nucleus accumbens, anterior cingulate cortex DLPFC; Gene Set Enrichment Analyses; BrainInABlender.	No psychological assessment was used.
Kindler et al., 2020	MRI scanning in DLPFC.	*Ex vivo*: Reverse transcriptase–qPCR for: GFAP, Iba1, KAT I, KAT II, TDO, KMO, IL-1β, IL-6, IL-8, SERPINA3 in the DLPFC; ultra-high-performance liquid chromatography and gas chromatography–mass spectrometry for TRP, KYN, KYNA, QUINA. *In vivo*: ultra-high-performance liquid chromatography and gas chromatography–mass spectrometry for: TRP, KYN, KYNA, QUINA. CRP; qPCR for IL-6, IL-8, IL-18, Il-1β.	MRI scanning in DLPFC.	*Ex vivo*: Reverse transcriptase–qPCR for: GFAP, Iba1, KAT I, KAT II, TDO, KMO, IL-1β, IL-6, IL-8, SERPINA3 in the DLPFC; ultra-high-performance liquid chromatography and gas chromatography–mass spectrometry for TRP, KYN, KYNA, QUINA. *In vivo*: ultra-high-performance liquid chromatography and gas chromatography–mass spectrometry for: TRP, KYN, KYNA, QUINA. CRP; qPCR for IL-6, IL-8, IL-18, Il-1β	Cognitive evaluation: PANSS. WAIS-III, WTAR, LSN, LM I AND LM II from WMS-R, COWAT, TMT-A.
Zhang et al., 2020	No neurobiological analyses were performed.	Brain tissue from DLPFC, anterior cingulate cortex; quantitative PCR for: ALDH1L1, GFAP, GLT1, GS, S100b.	No neurobiological analyses were performed.	Brain tissue from DLPFC, anterior cingulate cortex; quantitative PCR for: CD68, C-X3-C motif, CX3CR1, HLA-DRA, Iba1, P2RY12, TREM2, TSPO.	No psychological assessment was used.
Corley et al., 2021	Structural MRI.	Genome-wide association study; gene-set analysis restricted to genes that showed increase within microglial cells, comparison with two additional gene-sets from genes expressed within neuronal and astroglial cells; gene enrichment analysis: MAGMA.	Structural MRI.	Genome-wide association study; gene-set analysis restricted to genes that showed increase within microglial cells, comparison with two additional gene-sets from genes expressed within neuronal and astroglial cells; gene enrichment analysis: MAGMA.	Cognitive evaluation: WAIS-III, WTAR, LSN, CANTAB, WMS-III, PAL.
Shimamoto-Mitsuyama et al., 2021	Liquid chromatography with tandem mass spectrometry in anterior portion of corpus callosum and Brodmann area 8.	qPCR for target gene expression (GFAP).	Liquid chromatography with tandem mass spectrometry in anterior portion of corpus callosum and Brodmann area 8.	qPCR for target gene expression (CD68, CSF1R, AIF1).	No psychological assessment was used.
Uranova et al., 2021	Transmission electron microscopy, morphometry performed in layer 5 of the PFC.	No genetic or epigenetic analyses were performed.	Transmission electron microscopy, morphometry performed in layer 5 of the PFC.	No genetic or epigenetic analyses were performed.	No psychological assessment was used.
Di Biase et al., 2022	MRI scanning, cortical thickness measurement; analysis of cortical thickness (CTh) range deviation in schizophrenia patients.	Cell type transcriptional maps: astrocytes, endothelial, microglial, OPCs, excitatory neurons, inhibitory neurons, oligodendrocytes; interregional levels of cell type-specific gene expression were correlated across the individual deviations in regional CTh estimates.	MRI scanning, cortical thickness measurement; analysis of cortical thickness range deviation in schizophrenia patients.	Cell type transcriptional maps: astrocytes, endothelial, microglial, OPCs, excitatory neurons, inhibitory neurons, oligodendrocytes; interregional levels of cell type-specific gene expression were correlated across the individual deviations in regional CTh estimates.	No psychological assessment was performed.
Zong et al., 2023	DTI, T1 weighted MRI, before and after treatment; construction of morphometric similarity network.	Transcriptional profiles from Allen Human Brain Atlas; enrichment analysis; virtual histology of longitudinal MSN variation: overlap the PLS analysis loading genes with the gene set of each cell class (astrocytes); blood sample collection and genomic methylation levels analysis.	DTI, T1 weighted MRI, before and after treatment; construction of morphometric similarity network.	Transcriptional profiles from Allen Human Brain Atlas; enrichment analysis; virtual histology of longitudinal MSN variation: overlap the PLS analysis loading genes with the gene set of each cell class (microglia); blood sample collection and genomic methylation levels analysis.	Cognitive evaluation: PANSS. TMT.

**TABLE 7:** Summary of the results of studies performing analyses on both astrocytes and microglia.

Results	Astrocytes		Microglia		Psychological assessment
References	Neurobiological and morphological analyses	Genetic and epigenetic analyses	Neurobiological and morphological analyses	Genetic and epigenetic analyses	
Hagenauer et al., 2018	No neurobiological results were detected	No difference in astrocytes levels was detected in the PFC of schizophrenia patients compared to healthy controls.	No neurobiological results were detected.	No difference in microglia levels was detected in the PFC of schizophrenia patients compared to healthy controls.	No psychological evaluation was performed.
López-González et al., 2019	No neurobiological results were detected	No altered expression levels for GFAP were detected in schizophrenia patients compared to healthy controls.	No neurobiological results were detected.	Significant decrease of CD68 mRNA in the DLPFC of schizophrenia patients compared to controls; correlation between the expression levels of inflammatory genes and CD68 in schizophrenia patients; no difference was found in AIF1 expression levels.	No significant association were found between any altered gene and the severity of negative symptoms.
Karpiński et al., 2020	No neurobiological results were detected	Up-regulation of genes specific to astrocytes, interleukin-23, RUNX1, RUNX3 in DLPFC and PC of schizophrenia patients; down-regulation of RUNX2 in DLPFC and of GABA in DLPFC and PC.	No neurobiological results were detected.	Increase level of microglia in DLPFC in schizophrenia patients; decrease level of microglia in PC in schizophrenia patients.	No psychological evaluation was performed.
Kindler et al., 2020	No neurobiological results were detected	Positive correlation between GFAP and KAT I mRNA in schizophrenia patients and healthy controls; a feeble association between GFAP and KAT II in schizophrenia patients.	No neurobiological results were detected.	No significant correlation between Iba1 and KMO mRNA in schizophrenia patients and healthy controls.	Decrease in cognitive domain scores in schizophrenia patients; high cytokines subgroup showed superior premorbid estimates, worse attention scores compared to low cytokine subgroup.
Zhang et al., 2020	No neurobiological results were detected	Increase of gene ALDH1L1 in DLPFC and anterior cingulate cortex of schizophrenia patients compared to controls; increase of ALDH1L1 and glutamine synthetase in schizophrenia-NS compared to schizophrenia-S and healthy controls.	No neurobiological results were detected.	In DLPFC no changes were detected in microglia-related gene expression; In anterior cingulate cortex was observed an increase of CX3CR1 mRNA in schizophrenia-S compared to schizophrenia-NS (not significant when compared to controls), an increase in P2RY12 mRNA in schizophrenia-S compared to schizophrenia-NS, a decrease of TREM2 mRNA in schizophrenia-NS compared to schizophrenia-S.	No psychological evaluation was performed.
Corley et al., 2021	No neurobiological results were detected.	No enrichment was detected for the astrocyte gene-set.	No neurobiological results were detected.	No significant enrichment was detected for microglial gene-set; in replication sample, total gray matter volume mediated the association between microglial schizophrenia polygenic-score and general cognitive ability.	Reverse association between microglial schizophrenia-polygenic score and performance IQ, full-scale IQ, episodic memory.
Shimamoto-Mitsuyama et al., 2021	No neurobiological results were detected	No significant difference in expression level of GFAP was found in schizophrenia patients compared to controls.	No neurobiological results were detected.	Downregulation of AIF1 and CD68 in the corpora callosa of schizophrenia patients compared to controls; strong correlation between expression level of microglial marker genes and IL1B, TGFB1 and CSF1R.	No psychological evaluation was performed.
Uranova et al., 2021	Focal lysis, membranous debris in the cytoplasm of astrocytes, dystrophic astrocytes adjacent to microglia in schizophrenia patients.	No genetic or epigenetic results were detected.	Increased of microglial density in the schizophrenia patients compared to controls; dystrophy and hyperactivity of microglial cells; reduction in area, Vv and the number of mitochondria, an increase in area, Vv and the number of lipofuscin granules in schizophrenia patients.	No genetic or epigenetic results were detected.	No psychological evaluation was performed.
Di Biase et al., 2022	Schizophrenia patients showed more infra-normal deviations compared to controls in frontal, temporal, insular cortex, especially in the insula and rostral middle frontal cortex and caudal middle frontal gyrus.	Significant covariation of cortical thickness deviation with interregional expression levels of genes marking astrocytes, endothelial cells, OPCs, excitatory and inhibitory neurons in schizophrenia patients.	Schizophrenia patients showed more infra-normal deviations compared to controls in frontal, temporal, insular cortex, especially in the insula and rostral middle frontal cortex and caudal middle frontal gyrus.	No significant covariation between cortical thickness and interregional expression levels of genes marking microglia and oligodendrocytes.	No psychological evaluation was performed.
Zong et al., 2023	No neurobiological results were detected	MSN-related genes were involved in astrocytes, excitatory neurons, inhibitory neurons, endothelial cells, microglia cells oligodendrocytes; 174 overlapping genes for astrocytes.	No neurobiological results were detected.	MSN-related genes were involved in microglia cells, excitatory neurons, inhibitory neurons, astrocytes, endothelial cells, microglia cells oligodendrocytes; 80 overlapping genes for microglia.	Significant association between longitudinal alterations on the PANSS general psychopathology subscale and longitudinal changes in morphometric similarity network.

## 3 Results

### 3.1 Selected studies investigating astroglia and microglia in patients with a diagnosis of schizophrenia

**FIGURE 2:**
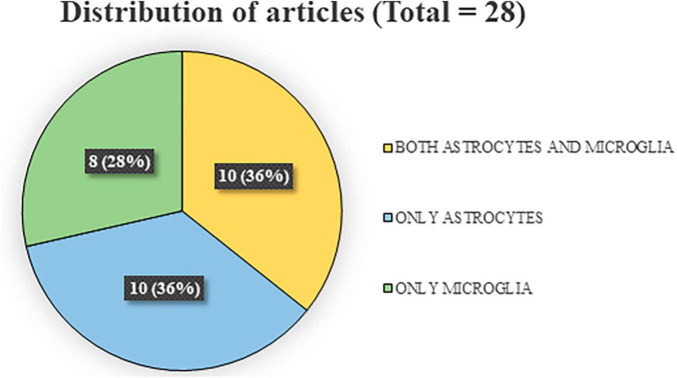
Distribution of articles according to the analyses conducted.

The research in the three databases produced a total of 1,358 articles, divided, respectively, in 338 out of PubMed search, 561 out of Scopus, and 459 out of Web of Science. After excluding 595 duplicates, 763 papers were screened by reading title and abstract and out of these 678 publications were discarded and 85 were selected for the full-text screening. After the screening, 57 articles were discarded as they did not match the inclusion criteria. The reasons for exclusion, along with their corresponding numbers, are reported in Figure 1. A total of 28 articles were used to write the systematic review. Of these articles, 20 focused on astrocyte analysis (Hagenauer et al., 2018; O’Donovan et al., 2018; López-González et al., 2019; Amoah et al., 2020; Chan et al., 2020; Karpiński et al., 2020; Kindler et al., 2020; Rodrigues-Amorim et al., 2020; Zhang et al., 2020; Corley et al., 2021; Jeon et al., 2021; Ranganathan et al., 2021; Shimamoto-Mitsuyama et al., 2021; Uranova et al., 2021; Di Biase et al., 2022; Endres et al., 2022; Huang et al., 2022; Pinjari et al., 2022; Schmitt et al., 2022; Zong et al., 2023), and 18 focused on microglia analysis (Hagenauer et al., 2018; López-González et al., 2019; Adorjan et al., 2020; Karpiński et al., 2020; Kindler et al., 2020; Petrasch-Parwez et al., 2020; Uranova et al., 2020, 2021; Zhang et al., 2020; Corley et al., 2021; Hill et al., 2021; Jiang et al., 2021; Shimamoto-Mitsuyama et al., 2021; Di Biase et al., 2022; Vikhreva and Uranova, 2022; Jenkins et al., 2023; Yu et al., 2023; Zong et al., 2023). Importantly, on the total of the researches concerning astroglia and microglia measures, 10 evaluated both aspects. Finally, 7 studies also included psychological (cognitive or emotional) assessments. Figure 2 depicts the percentages of the divisions relative to the total number of articles.

### 3.2 Neurobiological and morphological analyses on astroglia

The methodologies and results concerning investigations on astroglia in patients with schizophrenia are summarized in Tables 2, 3.

#### 3.2.1 *Ex vivo*

Based on the literature screening, only 1 article performed *ex vivo* neurobiological and morphological analyses on astrocytes (Schmitt et al., 2022). In this *post-mortem* investigation, a stereological approach was applied to analyze number and density of astrocytes, oligodendrocytes, and neurons in the hippocampal *Cornu Ammonis* (CA) 4, as well as of granular neurons in the dentate gyrus (DG) of left and right hemispheres in patients with schizophrenia and healthy controls. The results indicated a decrease in number and density of oligodendrocytes in the bilateral CA4 of patients. However, the number and density of astrocytes and neurons, as well as mean volume of CA4 and the DG, did not differ between patients and controls, even after adjustments for variables, such as *post-mortem* interval and age. The results suggest a potential deficit in oligodendrocyte maturation or a loss of mature oligodendrocytes, implicating an impaired myelination in schizophrenia.

#### 3.2.2 *In vivo*

Regarding *in vivo* neurobiological and morphological analyses of astrocytes, the literature screening produced 6 studies (Rodrigues-Amorim et al., 2020; Jeon et al., 2021; Ranganathan et al., 2021; Endres et al., 2022; Huang et al., 2022; Pinjari et al., 2022), 3 of whom evaluated the astrocytes by performing analyses of the glial fibrillary acidic protein (GFAP) marker, a structural component of astrocytes cytoskeleton (Rodrigues-Amorim et al., 2020; Ranganathan et al., 2021; Huang et al., 2022). GFAP is the protein of intermediate filament in astrocytes and it is commonly used as a hallmark of reactive astrocytes, contributing to the neuroplasticity by dynamic processes, such as the morphology of dendrites, synapsis formation, and neuronal migration and differentiation (Moehle et al., 2012; Kapitein and Hoogenraad, 2015). Furthermore, in the case of reactive astrogliosis, the reactive astrocytes show altered expression of many genes and the *GFAP* gene upregulation that coincides with changes in the immune response and neural communication (Hol and Pekny, 2015). Rodrigues-Amorim et al. (2020) focused on three structural proteins, such as GFAP, β-III tubulin (protein of microtubule of dendrites and axons), and neurofilament light chain (Nf-L) (structural protein exclusive of neurons) that are associated with neurological impairment and clinical progression of neurodegenerative disorders, with the aim of establishing a pattern of neurodegeneration in patients with schizophrenia. Estimating plasma protein levels through quantitative immunoblotting and ELISA kit, the authors showed higher GFAP, β-III tubulin, and Nf-L levels in patients with schizophrenia compared to controls, especially in the subgroup of chronic clozapine-treated patients, suggesting that the three structural proteins may be potential biomarkers to indirectly measure the neuronal damage in schizophrenia. Similarly, Ranganathan et al. (2021) carried out nanoparticle tracking analysis in plasma of patients with schizophrenia and healthy controls, to determine the size and concentration of circulating exosomes. Exosomes are small secretory microvesicles that influence neuronal and glial function via their microRNA (miRNA) cargo, positioning them as a novel and effective method of cell-to-cell communication with important implications for brain disease. Through Western Blot analyses, the authors also examined CD9 exosomal membrane marker and GFAP level, showing a higher concentration of exosomal GFAP in the plasma from patients compared to healthy controls. Furthermore, Huang et al. (2022) measured the levels of GFAP and S100β—another astrocytic marker—as well as IL-6, nerve growth factor (NGF), brain derived neurotrophic factor (BDNF), and TNF-α in the peripheral blood of patients with schizophrenia and healthy controls. Contrary to previous studies, their results showed no significant difference in GFAP concentration between groups, whereas a higher concentration of S100β, IL-6, NGF, and TNF-α was detected in patients’ group compared to control group. The levels of astrocytic marker S100β, P-selectin (cell adhesion molecule involved in periphery-to-brain communication pathway), and IL-6 were also measured in the plasma samples of patients with schizophrenia and controls (Pinjari et al., 2022). In the patients, P-selectin levels positively correlated with S100β and IL-6 levels, supporting the hypothesis that in schizophrenia peripheral immune activation may be associated to neuroinflammation, resulting thus in astrocytic activation. In another study (Endres et al., 2022), serum and cerebrospinal fluid samples from patients with schizophreniform and affective syndromes were examined for immunoglobulin G anti-CNS autoantibodies using tissue-based assays with indirect immunofluorescence, as part of an expanded routine clinical practice. Anti-CNS immunoglobulin G autoantibodies were identified in 18% of patients (serum 9%, CSF 18%), and among the five principal patterns that emerged one pattern was related to antibodies against astrocytes (serum 1%, CSF 1%). Finally, Jeon et al. (2021) focused on an indirect measurement of astrocytic function, by analyzing myo-inositol through 7-Tesla magnetic resonance spectroscopy at the dorsal part of anterior cingulate cortex (ACC). Specifically, myo-inositol is a molecule highly expressed in astrocytes (Brand et al., 1993), and reactive astrogliosis can cause its increased value, just as GFAP marker. For this reason, patients were scanned at baseline and after 6 months of antipsychotic treatment. At baseline, subjects affected by first-episode schizophrenia had lower levels of myo-inositol than healthy controls, while no difference was detected at follow-up, after antipsychotic treatment. Lower than normal levels of myo-inositol may indicate impaired astroglial activity, vulnerability for redox imbalance, excitotoxicity, and inappropriate astroglia-mediated inflammatory defense in the face of adversity. This supports the possibility of a putative astroglial deficit predating the first presentation of psychosis, likely of early developmental or of inflammatory origin (Dietz et al., 2020; d’Almeida et al., 2020), but showing a trend of reversal with early intervention.

### 3.3 Neurobiological and morphological analyses on microglia

The methodologies and results concerning investigations on microglia in patients with schizophrenia are summarized in Tables 4, 5.

#### 3.3.1 *Ex vivo*

Based on literature screening, 5 articles performed *ex vivo* neurobiological and morphological analyses on microglia (Adorjan et al., 2020; Petrasch-Parwez et al., 2020; Uranova et al., 2020; Hill et al., 2021; Vikhreva and Uranova, 2022). Transmission electron microscopy and morphometry were conducted on microglia and adjacent oligodendrocytes in layer 5 of the PFC extracted from *post-mortem* brains of two groups of subjects with schizophrenia, exhibiting predominantly positive or negative symptoms, and healthy controls (Uranova et al., 2020). The qualitative analysis revealed microglial activation and dystrophic alterations in both microglia and oligodendrocytes, situated in close proximity to each other in both clinical subgroups when compared to controls. Furthermore, both clinical subgroups exhibited a reduction in volume density and number of mitochondria, alongside an increase in the number of lipofuscin granules in oligodendrocytes and microglia. In microglia, volume density of lipofuscin granules as well as volume density and number of vacuoles of endoplasmic reticulum were significantly increased in the clinical subgroup exhibiting predominantly positive symptoms, compared to controls. In this clinical subgroup, the area of the nucleus of microglial cells showed negative correlations with age and age at illness onset. The findings suggest that microglial dystrophy may contribute to oligodendrocyte dystrophy, particularly in subjects with positive symptoms during relapse, and that mitochondria in both microglia and oligodendrocytes emerge as potential targets for treatment strategies in schizophrenia. Very similarly, *post-mortem* electron microscopy morphometric assessment was conducted on the layer 5 of PFC of patients with chronic attack-like progressive or continuous schizophrenia, in comparison to healthy controls (Vikhreva and Uranova, 2022). Various parameters, such as the numerical density of microglia, microglial soma and nucleus areas, nucleus-cytoplasm ratio, volume fraction, area and number of mitochondria, vacuoles of endoplasmic reticulum, and lipofuscin granules were estimated. The results indicated decreases in the volume fraction and number of mitochondria in both groups of patients, along with increases in these parameters for lipofuscin granules compared to the control group. Furthermore, patients with attack-like progressive schizophrenia exhibited increased microglial density and vacuole area, compared to the controls. Specifically, microglial density was higher in the subgroup of young patients (≤50 years) compared to the subgroup of older controls (>50 years). Furthermore, young patients displayed increases in microglial soma and nuclear areas compared to elderly controls, elderly patients with attack-like progressive schizophrenia, and young patients with continuous schizophrenia. Notably, in the patients with attack-like progressive schizophrenia, contrary to patients with continuous schizophrenia, microglial soma and nuclear areas, as well as the number of mitochondria, correlated negatively with age, while the area of lipofuscin granules correlated positively with age and disease duration. Thus, chronic attack-like progressive schizophrenia is characterized by increased microglial reactivity at a young age, accompanied by dystrophic changes in microglia that progress with age and disease duration. In contrast, continuous schizophrenia is associated with decreased microglial reactivity and non-progressive dystrophic changes.

One mechanism by which neuron-microglial communication occurs is via fractalkine signaling, and in this framework Hill et al. (2021) quantified microglia and analyzed the association between the chemokine fractalkine (CX3CL1) and its microglial receptor (CX3CR1), in *post-mortem* brain tissue derived from dorsolateral prefrontal cortex (DLPFC) of patients with schizophrenia, or bipolar disorder, and matched controls. While CX3CR1 levels did not differ between both clinical groups and control groups, fractalkine levels were lower in patients with schizophrenia, compared to controls, and unchanged in patients with bipolar disorder. A negative association was observed between fractalkine protein levels and lifetime antipsychotic dose in patients with schizophrenia. Presence of undifferentiated or paranoid schizophrenia subtypes, prescribed antidepressants or mood stabilizers, and cause of death (suicide or other cause) did not impact levels of fractalkine and CX3CR1.

In contrast to these data, other studies have not found any difference (Adorjan et al., 2020; Petrasch-Parwez et al., 2020). By quantifying ionized calcium-binding adapter molecule 1 (Iba1) and transmembrane protein 119 (TMEM119)-immunopositive cells in striatal and cortical neurons, Adorjan et al. (2020) assessed the state of microglial activation in schizophrenia. They showed no difference between patients and controls, reporting that amoeboid forms with large cell bodies characteristic of activated microglia were occasionally seen in both groups. Accordingly, Petrasch-Parwez et al. (2020) revealed no differences in microglial density in the anterior midcingulate cortex (aMCC) of patients with schizophrenia and healthy controls. Nevertheless, the authors found a significant lateralization of microglial density to the right aMCC in patients with schizophrenia.

#### 3.3.2 *In vivo*

Regarding *in vivo* neurobiological and morphological analyses of microglia, the literature screening did not produce any result consistent with inclusion criteria.

### 3.4 Neurobiological and morphological analyses on both astroglia and microglia

The methodologies and results concerning investigations on astroglia and microglia in patients with schizophrenia are summarized in Tables 6, 7.

#### 3.4.1 *Ex vivo*

One study investigated the activation of both astroglia and microglia as potential contributors to the development of schizophrenia, performing *ex vivo* neurobiological and morphological analyses (Uranova et al., 2021). Transmission electron microscopy and morphometry techniques were employed to assess microglial density and ultrastructural parameters in layer 5 of the PFC from *post-mortem* brain samples of individuals with chronic schizophrenia and healthy controls. Only in patients, the researchers found the presence of dystrophic (containing damaged mitochondria) and hyperreactive (containing many vacuoles) astrocytes adjacent to microglia, characterized by focal lysis and membranous debris in the cytoplasm. The same result was found for oligodendrocytes and neurons. Microglia morphometric analysis demonstrated a 20% increase in microglial density in patients, particularly in the younger ones (≤50 years old), those with a shorter duration of illness (≤26 years), and those characterized by early age at the disease onset (≤21 years), compared to controls. Furthermore, a decrease in area, volume fraction, and number of mitochondria, as well as an increase in area, volume fraction, and number of vacuoles and lipofuscin granules were found in patients compared to the healthy subjects, especially in both young and elderly patients compared to their respective controls. The volume fraction and the number of lipofuscin granules were positively correlated with age and duration of the disease in patients. Apoptotic microglia engulfed by dystrophic astrocytes supports the evidence that microgliosis (associated with age, duration of illness, and age at the onset of the disease) indicates signs of toxicity in the PFC gray matter of patients with schizophrenia.

#### 3.4.2 *In vivo*

Regarding *in vivo* neurobiological and morphological analyses of both astroglia and microglia, the literature screening did not produce any result consistent with inclusion criteria.

### 3.5 Genetic and epigenetic analyses on astroglia

The methodologies and results concerning investigations on astroglia in patients with schizophrenia are summarized in Tables 2, 3.

#### 3.5.1 *Ex vivo*

Regarding *ex vivo* genetic and epigenetic analyses of astroglia, the literature screening produced 3 studies (O’Donovan et al., 2018; Amoah et al., 2020; Chan et al., 2020). O’Donovan et al. (2018) meticulously isolated enriched populations of pyramidal neurons and astrocytes from the DLPFC of patients with schizophrenia and control subjects, employing laser capture microdissection, and then examined the expression of components within the adenosine system, by using quantitative polymerase chain reaction (qPCR). Intriguingly, the investigation revealed notable alterations in the enriched populations of both astrocytes and neurons, encompassing various metabolic and catabolic pathways. Specifically, a reduction in mRNA levels of ectonucleoside triphosphate diphosphohydrolase-1 (ENTPD1) and ENTPD2 in the enriched populations of astrocytes was found, suggesting a modulation in the expression of genes that play a pivotal role in the regulation of ATP metabolism within astrocytes. These changes may contribute to a diminished availability of substrates necessary for the generation of adenosine.

Starting from the assumption that in the brain miRNAs can modulate multiple molecular functions from neurogenesis to neuronal differentiation, from circuitry establishment to plasticity (McNeill and Van Vactor, 2012), a recent investigation unveiled noteworthy findings through the application of mature miRNA profiling and quantitative real-time PCR (qRT-PCR) in the orbitofrontal cortex (OFC) of patients with schizophrenia, or bipolar disorder, and control subjects (Amoah et al., 2020). Specifically, an elevation in the mature miRNA levels of miR-223, an exosome-secreted miRNA known for its targeting of glutamate receptors, was found in the OFC of patients with schizophrenia or bipolar disorder who had a positive history of psychosis at the time of death. This upregulation was correlated positively with astrocyte-enriched inflammation-related mRNA SERPINA3 and negatively with its target genes coding for glutamate ionotropic receptor NMDA-type subunit 2B and glutamate ionotropic receptor AMPA-type subunit 2. Additionally, miR-223 was found to be enriched within astrocytes and highly expressed in both glial and neuronal exosomes. Its expression was also differentially regulated by antipsychotic treatment in neuronal and astrocytic cultures. Furthermore, intriguing correlations between changes in miR-223 levels and expression of inflammatory (positive association) and GABAergic genes (negative association) were demonstrated. Notably, the introduction of astrocytic exosomes to cortical neurons led to an increase in neuronal miR-223 expression and reversed by inhibiting miR-223 in astrocytes, suggesting that miRNA altered by psychosis and enriched in glial cells, potentially under the influence of antipsychotic drugs, was released via exosomes to suppress the expression of neuronal NMDA receptor genes. Finally, Chan et al. (2020) utilized methylome-wide association (MWAS) data in blood of patients with schizophrenia. By comparing the MWAS results with findings of three existing large-scale array-based methylation studies on schizophrenia, researchers found two most significant sites: the first one was located in the *MFN2* gene, which encodes mitofusin-2 that regulates Ca^2+^ transfer from the endoplasmic reticulum to mitochondria, and the second one in *ALDH1A2* gene, which encodes a key enzyme in the production of astrocyte-derived retinoic acid in the brain. Notably, retinoids are potent morphogens critical for neurodevelopment and broadly implicated in schizophrenia (Goodman, 1998).

#### 3.5.2 *In vivo*

Regarding *in vivo* genetic and epigenetic analyses of astroglia, the literature screening did not produce any result consistent with inclusion criteria.

### 3.6 Genetic and epigenetic analyses on microglia

The methodologies and results concerning investigations on microglia in patients with schizophrenia are summarized in Tables 4, 5.

#### 3.6.1 *Ex vivo*

Regarding *ex vivo* genetic and epigenetic analyses of microglia, the literature screening produced 4 studies (Adorjan et al., 2020; Hill et al., 2021; Jenkins et al., 2023; Yu et al., 2023). Employing qPCR technology, Jenkins et al. (2023) quantified transcript levels for complement components and microglia-specific phagocytic markers from *post-mortem* PFC of patients with schizophrenia and control subjects. The authors found markedly higher mRNA levels for C4, which marks synapses for phagocytosis by microglia, and for C1q, which initiates the classical complement pathway that includes C4, in the patients when compared to control subjects. Furthermore, in the same patients gene expression was upregulated for multiple microglia specific markers that enable phagocytic activity, including TAM receptor tyrosine kinases (Axl, MerTK) and lysosome-associated membrane glycoprotein CD68. Transcript levels for these markers of microglial phagocytosis were positively correlated with each other and with C4 mRNA levels in patients. Furthermore, higher mRNA levels for THIK1 (selectively expressed by microglia and regulating cytokine release in activated microglia) and lower mRNA levels for purinergic receptor P2Y12 (selectively expressed by microglia and scarcely expressed in activated microglia) were found in patients when compared to controls. In addition, mRNA levels for C3 or CR3 (which are additional components of the complement pathway), or for the microglia-specific markers triggering receptor expressed on myeloid cells-2 (TREM2) and its transmembrane binding partner (involved in microglial phagocytosis of spines), were not different between patients and non-psychiatric subjects. No effects of antipsychotic treatment on microglia-related gene expression were found. Analyzing the role of striatal interneurons and cortical neurons and their associations with inflammation in schizophrenia, Adorjan et al. (2020) reported no differences in the transcript levels of Iba1 and TMEM119 between patients and healthy controls. In parallel, carrying out droplet digital PCR to quantify mRNA levels for fractalkine, CX3CR1 and metalloproteinase 10 (ADAM10), Hill et al. (2021) did not show differences in the mRNA expressions in the *post-mortem* DLPFC of patients affected from schizophrenia, bipolar disorder, and healthy controls. Yu et al. (2023) also examined transcriptional profiles in the DLPFC of patients with schizophrenia and healthy controls through *post-mortem* RNA-sequencing to analyze sex-specific differences in gene expression. Using Gene Ontology Analysis and Principal Component Analysis, the researchers revealed sex-specific genes associated with schizophrenia: in the female group, schizophrenia-relevant genes were overexpressed in midbrain dopaminergic and GABAergic neurons and in PFC C3-microglia.

#### 3.6.2 *In vivo*

Regarding *in vivo* genetic and epigenetic analyses of microglia, the literature screening produced only 1 result consistent with inclusion criteria (Jiang et al., 2021). The authors utilized several datasets of patients with schizophrenia and healthy controls to analyze the association between gene component and structural brain component, employing also an independent *in vivo* cohort for a second validation. Their results showed a negative correlation between the PFC/insula structural component and microglia-related genes. In the validation cohort, one of the most contributing single nucleotide polymorphisms was located in microglial-related *TMEM119* gene.

### 3.7 Genetic and epigenetic analyses on both astroglia and microglia

The methodologies and results concerning investigations on astroglia and microglia in patients with schizophrenia are summarized in Tables 6, 7.

#### 3.7.1 *Ex vivo*

Based on the literature screening, 7 articles performed *ex vivo* genetic and epigenetic analyses on astrocytes and microglia (Hagenauer et al., 2018; López-González et al., 2019; Karpiński et al., 2020; Kindler et al., 2020; Zhang et al., 2020; Shimamoto-Mitsuyama et al., 2021; Di Biase et al., 2022). Two studies employed a method that utilizes the collective expression of cell type transcripts in brain tissue samples to predict cell type expression (Hagenauer et al., 2018; Di Biase et al., 2022). Hagenauer et al. (2018) compiled a database of transcripts that were specifically enriched in one of the primary brain cell types based on previous single-cell or purified cell-type transcriptomic experiments: astrocytes, microglia, endothelial cells, mural cells, immature and mature oligodendrocytes, interneurons, and projection neurons. Specific cell-type transcripts were identified using forebrain or cortical samples of subjects with schizophrenia, major depressive disorder, or bipolar disorder. No difference emerged in astrocyte and microglia indices in the PFC of patients with schizophrenia. In Di Biase et al. (2022), the authors employed gene expression data from *post-mortem* brains for transcriptional analysis of specific cortical regions and cell types: astrocytes, microglia, endothelial cells, oligodendrocytes, oligodendrocyte progenitors, and excitatory and inhibitory neurons. In individuals with schizophrenia classified in cell-based subtypes, the associations between cortical thickness deviation profile and interregional gene expression maps of specific cell types were investigated, proving that cortical thickness deviations covaried with interregional expression levels of genes marking astrocytes (but not microglia), as well as endothelial cells, oligodendrocyte progenitors, and excitatory and inhibitory neurons.

Transcriptome and gene-set enrichment analyses were also conducted based on seven datasets collecting gene expression data from associative striatum, hippocampus, anterior PFC, superior temporal cortex, middle frontal area, DLPFC, and parietal cortex (Karpiński et al., 2020). Applying a method to deconvolute RNA expression data and estimate indexes of brain cell types, the authors observed decreased indexes of astrocytes and neurons in patients with schizophrenia in comparison to controls, both in DLPF and parietal cortex. Furthermore, in the patients (compared to controls) the index of microglia was higher in DLPFC, while indexes of microglia and endothelial cells were lower in parietal cortex. The deregulation of twenty-one key genes, including astroglial and microglial markers, was also analyzed in *post-mortem* DLPFC of elderly subjects with chronic schizophrenia (López-González et al., 2019). No altered expression levels for astroglial marker GFAP and lower expression levels for microglia marker CD68 were found in patients with schizophrenia compared to controls. Very similarly, mRNA expression of GFAP, Iba1, IL-1β, IL-6, IL-8, SERPINA3, as well as of kynurenine aminotransferases (KAT I, KAT II, belonging to kynurenine pathway of tryptophan catabolism linking immune system activation with neurotransmitter signaling) was measured in the *post-mortem* DLPFC of subjects with schizophrenia and healthy controls (Kindler et al., 2020). Results showed a positive correlation between GFAP and KAT I mRNA expression in both patients and controls, and a weaker correlation between GFAP and KAT II mRNA expression only in patients. Finally, investigating whether suicidal and non-suicidal patients with schizophrenia differ from each other in terms of glial gene expression in the PFC, qPCR was performed on isolated gray matter of the DLPFC and ACC, using a panel of common markers for astrocytes, microglia and oligodendrocytes in patients with schizophrenia who died from either suicide or from natural causes and who were compared to healthy controls (Zhang et al., 2020). Namely, mRNA expression of 16 glia-related markers including those for astroglia (aldehyde dehydrogenase 1 family member L1–ALDH1L1, and GFAP) and microglia (CX3CR1, Iba1, P2Y12 and TREM2) was quantified. In DLPFC and ACC, the expression of the astrocytic ALDH1L1 marker was higher in patients (especially who committed suicide) compared to controls. In parallel, in ACC the mRNA expression of microglial CX3CR1 and P2RY12 markers was higher in suicidal patients compared to no-suicidal patients, and TREM2 expression was lower in no-suicidal patients. The findings emphasize the heterogeneity in glia gene alterations in patients with schizophrenia in relation to suicidal conduct.

Finally, by using liquid chromatography coupled to tandem mass spectrometry and qPCR analyses, Shimamoto-Mitsuyama et al. (2021) carried out *post-mortem* investigations of the lipid contents of the *corpus callosum* from patients with schizophrenia and healthy controls, revealing low gene expression levels of microglial markers and colony-stimulating factor 1 receptor (known to regulate the density of microglia) in patients, but the same levels of astroglial (GFAP) markers between patients and controls. Genes associated to Iba1 (in the cited paper reported as allograft inflammatory factor 1–AIF1) and CD68 were downregulated in patients with schizophrenia compared to controls. The impaired schizophrenia-related expression of microglial Iba1 marker, found in the *corpus callosum*, was confirmed in the frontal cortex of the patients with schizophrenia. Gene expression analyses of inflammatory cytokines (such as IL-1β, IL-6, and TNF-α) revealed that the expression levels of IL-1β were significantly lower in patients than controls. Although inflammation is a major topic in schizophrenia research, these results show signs of anti-inflammatory reaction in patients with schizophrenia.

#### 3.7.2 *In vivo*

Regarding *in vivo* genetic and epigenetic analyses of astroglia and microglia, 2 articles deal with omic-investigations (Corley et al., 2021; Zong et al., 2023). Namely, Zong et al. (2023) performed an imaging-transcriptomic-epigenetic analysis to test whether omics data and cortical connectivity organization, measured by using morphometric similarity network (MSN), could be biomarkers to predict treatment outcomes and cognitive deficits in the early phase of schizophrenia. Patients, before and after 8-week risperidone monotherapy, and healthy controls underwent magnetic resonance imaging (MRI) and diffusion tensor imaging (DTI) protocol for MSN data investigations. In parallel, peripheral blood samples were collected for genomic DNA methylation status examinations. To identify MSN variations-related genes, it was performed a virtual histology with the gene set of specific cell classes: astrocytes, microglia, oligodendrocytes, endothelial cells, oligodendrocyte progenitors, and excitatory and inhibitory neurons. From enrichment analysis emerged that longitudinal alterations in MSN after the treatment were primarily enriched in neurobiological, immunologic, metabolic, and cognitive processes. Furthermore, 174 genes were highlighted in astrocytes after the treatment with the antipsychotic drug. Finally, in patients with a diagnosis of schizophrenia or other psychotic disorders compared with healthy participants, Corley et al. (2021) performed a gene-set analysis restricted to genes associated to astroglia cells, comparing them to gene-sets from genes associated to microglia and neuronal cells. Using a recent tool for gene-set analysis, it was demonstrated that the astroglia and microglia gene-sets were not enriched for genes associated with schizophrenia risk.

### 3.8 Psychological correlates

Based on the literature screening, 7 articles reported psychological (cognitive and emotional) assessment (López-González et al., 2019; Kindler et al., 2020; Rodrigues-Amorim et al., 2020; Corley et al., 2021; Jeon et al., 2021; Ranganathan et al., 2021; Zong et al., 2023), 6 of whom utilized the Positive and Negative Syndrome Scale (PANSS) (López-González et al., 2019; Kindler et al., 2020; Rodrigues-Amorim et al., 2020; Jeon et al., 2021; Ranganathan et al., 2021; Zong et al., 2023), a widely used scale, validated for measuring severity and prevalence of positive or negative symptoms in schizophrenia (Opler et al., 2017). Investigating the schizophrenia-relevant protein biomarkers in plasma exosomes derived from subjects affected by schizophrenia and healthy controls, Ranganathan et al. (2021) administered PANSS to patients and concluded that the psychopathological symptomatology did not correlate with concentration of astroglial marker GFAP in circulating exosomes. Similarly, Rodrigues-Amorim et al. (2020) administered PANSS to patients with first-episode schizophrenic psychosis or chronic schizophrenia, and showed that the positive and negative subscale scores of two groups of patients were not significantly different, even if in the two groups a significantly different plasma GFAP level was found. Zong et al. (2023) administered PANSS subscales and Trail Making Test (TMT)–which examines visual-motor coordination and attention (TMT-A) and task switching (TMT-B) to drug-naïve first-episode schizophrenia patients, before and after risperidone treatment, who in parallel were submitted to neuroimaging and omic evaluations. The findings showed an association between longitudinal changes in cortical connectivity organization after treatment and the longitudinal alterations on the PANSS general psychopathology subscale. In a *post-mortem* study evaluating the possible deregulation of genes involved in immune system and its association with *ante-mortem* PANSS evaluations, López-González et al. (2019) found no significant association between severity of negative symptoms and any altered gene. Furthermore, in a longitudinal investigation of microglial integrity status, quantified by ACC myo-inositol, Jeon et al. (2021) scored patients affected from first-episode schizophrenia and healthy controls on PANSS, Social and Occupational Functioning Assessment Scale (SOFAS), and Calgary Depression Scale for Schizophrenia (CDSS). Findings highlighted that myo-inositol concentration correlated negatively with PANSS scores, weakly with SOFAS scores, and not at all with CDSS score. More extensively, Kindler et al. (2020) evaluated cognitive domains of verbal memory, language, working memory, processing speed, and perceptual organization in chronic schizophrenia or schizoaffective patients and healthy controls, administering in addition to PANSS and TMT-A, also the Wechsler Adult Intelligence Scale-Third Edition (WAIS-III), Wechsler Test of Adult Reading (WTAR), WAIS-III Letter-Number Sequencing (LNS) test, Logical Memory I and II, Verbal Memory tests of the Wechsler Memory Scale-Revised (WMS-R), the Controlled Oral Word Association Test (COWAT), and F-A-S verbal fluency test. Blood plasma samples were collected from each participant submitted to cognitive assessment and, after, to MRI scanning. Results indicated a significant reduction of cognitive domain scores in patients with schizophrenia compared to controls, as well as a decrease in DLPFC volumes. Moreover, dividing patients according to their high and low levels of cytokines, the authors showed that the patients characterized by high cytokines levels had worse scores in premorbid intellectual and attentive evaluations compared to patients characterized by low cytokines levels.

Finally, Corley et al. (2021) administered WAIS-III, WTAR, LSN test, Spatial Working Memory task and Paired Associations Learning (PAL) task from the Cambridge Automated Neuropsychological Test Battery to patients with schizophrenia, to investigate the association between cognitive domain alterations and schizophrenia risk-associated glia gene expression. Results indicated no association between schizophrenia-related polygenic astroglia and microglia score and severity of symptoms. However, schizophrenia-related microglial polygenic score was inversely associated to scores in IQ performance, IQ full-scale, and episodic memory. In a replication sample, total gray matter volume mediated the association between microglial polygenic score and episodic memory performances.

## 4 Discussion

Astroglial and microglial cells have been implicated in schizophrenia, yet the precise glia-associated mechanisms in the pathophysiology of disease remain poorly understood. This is primarily due to conflicting and scattered results in studies that have measured these glial populations by employing various methods, ranging from morphological analyses to epigenetic investigations. This complexity is further exacerbated from the inherently intricate clinical picture of the schizophrenia. In general, astrocytes play vital roles in maintaining the redox balance in the brain (Bélanger et al., 2011), clearing extracellular glutamate from synaptic space (Schousboe et al., 2014), maintaining synaptic integrity, and supporting myelination (Volterra and Meldolesi, 2005; Bélanger and Magistretti, 2009), all critical processes in the mechanistic pathways suspected in schizophrenia. In the present systematic review, many *post-mortem* and *in vivo* neurobiological and morphological studies conducted on individuals with schizophrenia suggest an astrocyte overactivation (Ranganathan et al., 2021; Endres et al., 2022; Huang et al., 2022; Pinjari et al., 2022). Namely, hyperreactive and dystrophic astrocytes adjacent to microglia, oligodendrocytes, and neurons, were characterized by focal lysis and membranous debris in the cytoplasm, indicating signs of toxicity (Uranova et al., 2021). Furthermore, structural proteins such as GFAP, component of astrocytes cytoskeleton and indicative of reactive astrocytes, appear to significantly influence schizophrenia (Rodrigues-Amorim et al., 2020), by altering neuroplasticity through dynamic processes that involve dendrite morphology, synapsis formation, neuronal migration and differentiation. Moreover, the structural proteins, detectable in the peripheral samples, may serve as potential biomarkers for indirectly measuring neuronal damage, especially in the prodromal stages of schizophrenia, as suggested by the higher concentration of exosomal GFAP in the plasma of patients in comparison to healthy controls (Ranganathan et al., 2021).

Throughout brain development phases, microglial cells closely communicate with the newborn neurons, being sensitive and responsive to neurochemical signs, exerting a crucial modulatory role in the formation/maturation of neurons and neuronal circuits, contributing to brain homeostasis. Microglial cells are also able to mount an inflammatory response to insults, and perform synaptic pruning, which involves the phagocytosis of synapses (Paolicelli et al., 2011; Schafer et al., 2012). Notably, an aberrant synaptic pruning occurs in schizophrenia (Germann et al., 2021; Parellada and Gassó, 2021). Patients, especially those exhibiting predominantly positive symptoms, displayed microglial activation and dystrophic alterations in the PFC (Uranova et al., 2020, 2021). Namely, it is reported that younger patients exhibited increased microglial density, with heightened reactivity at a young age (in the early phase of disease), and dystrophic changes progressing with age and disease duration (in the chronic phases). Furthermore, chronic attack-like progressive schizophrenia was characterized by increased microglial reactivity at young age, accompanied by dystrophic changes in microglia that progress with age and disease duration, while continuous schizophrenia was associated with decreased microglial reactivity and non-progressive dystrophic changes (Vikhreva and Uranova, 2022). Thus, the presence of hyperactivated and apoptotic microglia engulfed by hyperactivated and dystrophic astrocytes supports the evidence that microgliosis and astrogliosis in patients with schizophrenia, associated with age, duration of illness, and age at disease onset, are signs of toxicity that may be precipitating factors for synaptic pruning.

However, conflicting findings have been reported in other *post-mortem* and *in vivo* neurobiological and morphological studies, in which the overactivation of astroglia (Jeon et al., 2021; Schmitt et al., 2022) and microglia (Adorjan et al., 2020; Petrasch-Parwez et al., 2020) was not observed in various brain regions of patients with schizophrenia compared to controls. To clarify the discrepancies in the role of astroglial and microglial activation, along with morphological changes, in the context of schizophrenia the omic-studies conducted to explore glia-associated genetic and epigenetic aspects are crucial. Each omic-study provided insight into different mechanisms potentially linked to the pathophysiology of schizophrenia. Data revealed schizophrenia-related alterations in astrocytes associated with variations in metabolic and catabolic pathways in DLPFC (O’Donovan et al., 2018), upregulation of inflammation pathways and downregulation of genes related to glutamate receptors in OFC (Amoah et al., 2020), and involvement of the *ALDH1A2* gene that encodes an enzyme in the production of astrocyte-derived retinoic acid, potent morphogen critical for neurodevelopment (Chan et al., 2020). Furthermore, in individuals with schizophrenia, cortical thickness deviations covaried with interregional expression levels of genes marking astrocytes (Di Biase et al., 2022). These studies collectively shed light on the complex genetic and epigenetic landscape associated with astroglial involvement in schizophrenia, emphasizing potential links to ATP metabolism, miRNA regulation, and retinoid-related pathways.

Vague insight into microglia-related molecular mechanisms associated with schizophrenia have been gained by many *ex vivo* studies employing genetic and epigenetic analyses of microglia. On one hand, multiple microglia-specific markers related to phagocytic activity and regulating cytokine release in activated microglia were upregulated in the PFC of patients with schizophrenia when compared to healthy subjects (Jenkins et al., 2023). On the other hand, contrasting results demonstrated low gene expression levels of microglial markers measured in the corpus callosum (Shimamoto-Mitsuyama et al., 2021), parietal cortex (Karpiński et al., 2020), and DLPFC (López-González et al., 2019) of patients with schizophrenia. These results along with those related to no enrichment for genes associated with schizophrenia risk in microglia gene-sets (Hagenauer et al., 2018; Adorjan et al., 2020; Corley et al., 2021; Hill et al., 2021; Di Biase et al., 2022) suggest potential anti-inflammatory or no-inflammatory reactions in the context of schizophrenia. A part of these studies reported also a lack of relationship (Hagenauer et al., 2018; López-González et al., 2019), or even an inverse relationship (Karpiński et al., 2020) between astroglia-related genes and schizophrenia risk.

Sex-specific and suicide-related differences were also found. Examining transcriptional profiles in the DLPFC of patients with schizophrenia and healthy controls through *post-mortem* RNA-sequencing, it has been demonstrated that schizophrenia-relevant genes were overexpressed in midbrain dopaminergic and GABAergic neurons, as well as in PFC C3-microglia of females (Yu et al., 2023). Further, higher expression of the astrocytic marker ALDH1L1 was observed in the DLPFC and ACC of patients, especially those who committed suicide, compared to controls (Zhang et al., 2020). In parallel, in ACC the microglial markers CX3CR1 and P2RY12 were higher and the microglial marker TREM2 was lower in suicidal patients compared to non-suicidal patients. Again, such findings emphasize the heterogeneity in glia gene alterations in patients with schizophrenia, also in relation to sex or suicidal conduct.

Importantly, several studies have conducted psychological (cognitive and emotional) assessments in individuals with schizophrenia, utilizing various scales and tests. A breakdown of the key findings of the mentioned studies indicates the complex interplay among cognitive functioning, symptomatology, and underlying glial mechanisms in schizophrenia. Scattering results range from no association between schizophrenia-related polygenic astroglia and microglia score and symptom severity (Corley et al., 2021) to microglial polygenic score inversely associated with scores in IQ performance, IQ full-scale, and episodic memory performances (Corley et al., 2021). Even if no significant association between severity of negative symptoms and any altered gene was found (López-González et al., 2019), and even if psychopathological symptomatology did not correlate with the concentration of the astroglial marker GFAP in circulating exosomes (Ranganathan et al., 2021), the myo-inositol concentration (index of microglial integrity status) correlated negatively with PANSS scores (Jeon et al., 2021), and patients with high cytokine levels had worse scores in premorbid intellectual and attentive evaluations (Kindler et al., 2020).

Overall, the alterations in neuron-glia communication are critical for understanding the pathological mechanisms of psychiatric disorders due to commitment of neurotransmitter systems, excitatory-inhibitory balance, neurotrophic state, and inflammatory response (Reis de Assis et al., 2021; Wartchow et al., 2023). Namely, in schizophrenia as well as in other neuropsychiatric disorders, as bipolar, mood and autism spectrum disorders, delirium, psychosis, and dementia, the activated microglia can induce the triad astrocyte reactivity, neuroinflammation, and oxidative stress (Schmitz et al., 2023). These changes in cellular responses and mechanisms causing neuronal death result in cognitive dysfunction, an often-aggravating factor of psychiatric disorders. Conversely, glioprotective factors attenuate glial damage by generating specific responses that can protect glial cells themselves and/or neurons, resulting in improved brain functioning and homeostasis (Quincozes-Santos et al., 2021). In this regard, cognitive functions impaired in schizophrenia primarily rely on excitatory synapses and dendritic spine density, crucial for neuroplasticity in cortical areas (Cahill et al., 2009). In turn, the density of synaptic connections and dendritic spines is partially regulated by astroglia and microglia which, beside their roles in neuroimmune and neuroinflammation regulation, are involved in the phagocytosis of synapses and dendritic spines (Tremblay et al., 2010; Paolicelli et al., 2011; Sekar et al., 2016; Sellgren et al., 2017; Filipello et al., 2018; Mallya et al., 2019). In schizophrenia, the overactivated astroglia and microglia may signify an immune system response to continuous dysregulation of processes related to neuroplasticity or an early reaction to early insults, possibly followed by astrogliosis and microgliosis. In both scenarios, astroglia and microglia play a crucial role in finely regulating synapse formation and elimination, contingent upon the accurate detection of neuronal/synaptic molecular signs. Astroglia and microglia undergo morphological adaptions based on their brain location and stimuli, an issue extensively studied in the context of mental diseases (Caetano et al., 2017; Simões-Henriques et al., 2020; Gaspar et al., 2021). Consequently, any alteration in astroglia and microglia has the potential to disrupt the normal course of development, influencing the number, maturation degree, and function of synapses/neurons, ultimately impacting behavior, cognitive functions, and health in the context of schizophrenia.

## 5 Limitation

Clinical findings are influenced by a myriad of confounding factors that must be carefully considered in future studies. These factors encompass individual variability, sex and age of onset, prevalence of positive or negative symptoms, and type and duration of pharmacological treatment for schizophrenia. Markers for astroglia and microglia used in *post-mortem* studies exhibit certain limitations, particularly in terms of selectivity. An overestimation of astroglia and microglia alterations may arise from the contribution of other cells stained by the same markers. Moreover, the impact of the cause of death, as well as the influence of the *post-mortem* interval, are crucial aspects to be considered in the design of future studies on schizophrenia-related changes in astroglia and microglia. Additionally, there are obvious technical limitations in evaluating astroglia and microglia, or their correlates, in living patients. It is imperative to reconsider and implement a variety of different markers, along with a clear definition of the outcomes to be analyzed. The interpretation of normal, increased, or decreased marker levels needs to be reexamined through a consensus evaluation, considering pathophysiological implications and their correlation with the clinical presentation of schizophrenia. Recently, *in vivo* microglial function in the brain has been quantified by using positron emission tomography (PET) and radiotracers specific for TSPO, a protein that is overexpressed by the activated microglia. Unfortunately, high TSPO levels compared to their relatively low physiological expression are not limited to neuroinflammation but also associated with other types of inflammation attributed to impaired cell metabolism, tumor proliferation, and autoimmune diseases. Consequently, the detection of neuroinflammation via TSPO detection may not be cell type-specific and may also involve non-inflammatory neuronal activity. This reduced specificity together with inability to differentiate between the detrimental and beneficial effects of inflammation, and inability to differentiate microglia and astroglia roles, might represent a limitation of TSPO as biomarker in brain imaging. An additional complicating factor for second-generation radioligands is their susceptibility to genetic polymorphisms responsible for differences in binding affinity that renders necessary prior genotyping of the patients. In spite of such restraints, PET with radioligands targeting the TSPO has been used to detect patterns of neuroinflammation *in vivo*, assess disease severity and progression, and therapeutic efficacy of anti-inflammatory drugs (Ottoy et al., 2018; Selvaraj et al., 2018; Veronese et al., 2018; De Picker et al., 2019; Schifani et al., 2019; Laurikainen et al., 2020; Conen et al., 2021; Da Silva et al., 2021; Marques et al., 2021).

Finally, the search for peripheral biomarkers reflecting neuropathological changes in schizophrenia is ongoing, even if obtaining relevant tissues in living patients proves challenging, and results have remained elusive. Exploring circulating extracellular vesicles represents a promising approach to identify potential peripheral biomarkers for schizophrenia. However, further studies are essential to elucidate the underlying mechanisms and explore potential therapeutic targets in astroglia and microglia for the treatment of schizophrenia.

## Data availability statement

The original contributions presented in the study are included in the article/supplementary material, further inquiries can be directed to the corresponding author/s.

## Author contributions

DL: Conceptualization, Project administration, Supervision, Visualization, Writing – original draft, Writing – review and editing. MPa: Data curation, Formal Analysis, Investigation, Methodology, Software, Validation, Visualization, Writing – original draft, Writing – review and editing. DD: Data curation, Formal Analysis, Investigation, Methodology, Resources, Software, Validation, Visualization, Writing – original draft, Writing – review and editing. AP: Data curation, Formal Analysis, Investigation, Methodology, Validation, Visualization, Writing – review and editing. DC: Conceptualization, Data curation, Supervision, Validation, Visualization, Writing – review and editing. MPe: Conceptualization, Supervision, Validation, Visualization, Writing – review and editing. CM: Funding acquisition, Supervision, Validation, Writing – review and editing. LP: Funding acquisition, Resources, Supervision, Validation, Writing – review and editing.
